# Development of Raw Materials and Technology for Pulping—A Brief Review

**DOI:** 10.3390/polym15224465

**Published:** 2023-11-20

**Authors:** Piwu Li, Yanpeng Xu, Liang Yin, Xiaoli Liang, Ruiming Wang, Kaiquan Liu

**Affiliations:** 1 State Key Laboratory of Biobased Material and Green Papermaking (LBMP), Qilu University of Technology (Shandong Academy of Sciences), Jinan 250353, China; piwuli@qlu.edu.cn (P.L.); 10431211110@stu.qlu.edu.cn (Y.X.); 10431211135@stu.qlu.edu.cn (X.L.); wrm@qlu.edu.cn (R.W.); 2 Key Laboratory of Shandong Microbial Engineering, College of Bioengineering, Qilu University of Technology (Shandong Academy of Sciences), Jinan 250353, China; 3 Gansu Engineering Technology Research Center for Microalgae, Hexi University, Zhangye 734000, China; yinl03@163.com

**Keywords:** pulp and paper, raw materials, wood, non-wood fibers, future trends

## Abstract

Paper is one of the most significant inventions in human civilization, which considerably advanced global cultural development. Pulping is a key step in the conversion of fiber raw materials into paper. Since its inception, pulping has rapidly evolved, continually adapting to technological advancements. Researchers are constantly investigating various types of raw materials for pulping. In this review, some of the materials employed in pulping are outlined, and the fiber content, pulping method, as well as the strength of wood and non-wood crop straw as pulping raw materials are analyzed and discussed. In addition, this review explores the effects of different materials under various pulping conditions and assesses the future trends in raw material selection for pulping while considering the current global environmental pressures.

## 1. Introduction

Paper comprises fibers that are bonded together through intermolecular forces [1]. Since the emergence of papermaking, the pulp and paper industry has been an integral part of the everyday lives of people, and paper has greatly contributed to civilization and economic development. The new era has largely increased the global demand for pulp and paper [2]. Besides improving the quality of people’s lives, paper has accelerated the spread of knowledge and promoted the development of science and technology.

According to 2022 statistics, the global pulping and papermaking industry completed a total output of 417 million t of paper and cardboard, with the United States and China accounting for ~78.2 and 85.87 million t, respectively. As a major pulp producer, China consumed 112.95 million t of pulp, imported 29.64 million t, and exported ~8.58 million t. Continuous efforts are required to discover raw materials for pulping and papermaking and improve the pulping technology and quality. The pulping and papermaking industry has recently entered a stable phase due to the huge demand for raw materials, the incomplete development of pulping materials, and the energy-intensive and polluting nature of traditional pulping processes [3].

The raw materials used in the pulping industry primarily include wood, non-wood, and recycled fibers. Although pulping methods have changed, primary raw materials, including plant and non-plant fibers, have remained the same. In recent years, traditional pulping materials have faced various restrictions, and environmental protection requirements have made the supply of wood raw materials insufficient. Therefore, finding new alternatives has emerged as a future development trend [4,5].

Alongside the need to find alternative raw pulping materials, problems associated with the pulping process must be addressed. Since the emergence of pulping technology 2000 years ago, pulping methods have undergone various changes. Moreover, the pulping process is complicated. Different raw materials and pulping methods produce different pulp characteristics and paper products, such as newsprint, corrugated paper, A4 paper, and special paper. Innovations include biological pulping using microorganisms, organic solvent pulping, and oxygen–alkali pulping using oxygen and chemical reagents [6]. The existence of each pulping process is meaningful, allowing for the selection or combination of suitable methods to produce economical and eco-friendly paper [7].

## 2. Pulping

**Figure 1:**
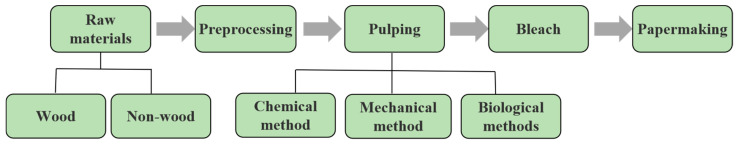
Flow of the pulping and papermaking process.

The pulping process is complex and generally divided into the following three stages: pulping, bleaching, and papermaking (Figure 1) [8]. Pulping is the separation of fibers from raw materials to obtain pulp, with wood mainly used as the raw material. Woody biosubstrates have accounted for over 90% of global pulp production for over a century [9].

The main pulping methods used during the pulping stage include chemical, mechanical, and biological pulping, as well as combinations of these to separate cellulose fibers from lignin and obtain coarse pulp that is used in papermaking. In the first pulping process, fibers were separated through mechanical grinding [10]. However, this process is energy-intensive and produces poor-quality coarse pulp. Chemical pulping emerged when Watt and Burgess found that lignin could be removed from wood using sodium hydroxide at high temperatures. Subsequently, the sulfite pulping and kraft processes were developed in 1866 and 1879, respectively [11]. Behrend’s concept of cooking materials before refining, which was proposed in 1869, laid the foundation for the development of modern mechanical and chemical mechanical pulping processes [12]. Compared to mechanical pulping, chemical pulping has a more considerable pulping effect and substantially reduces energy consumption; however, it affects the final pulping yield and increases the cost [13].

Biopulping primarily employs the growth of microorganisms, such as white rot fungi, to break down the lignin of raw materials, thus separating fibers and lignin. The pulping period of this method is longer compared with those of the chemical and mechanical methods, but the pulping effect is better than that of the mechanical method. Compared to chemical pulping, biopulping is inferior but has a high yield and low environmental impact [14]. Recent discussions have focused on combining these three pulping methods to balance energy consumption, pulp yield, and paper quality.

Bleaching, which is the process of removing residual pigments from fiber materials or products to achieve a certain degree of whiteness, can be mainly achieved through oxidation or reduction. The oxidation method uses oxidizing reagents, such as NaClO and H_2_O_2_, to turn colored molecules into colorless molecules, whereas the reduction method uses SO_2_ and other reagents to reduce colored molecules, causing less damage to fiber raw material [15]. Bleaching is the second step in the pulping process. The bleaching process is mainly divided into four parts, namely, the chlorine dioxide stage (D0), the antioxidant extraction stage (EOP), chlorine dioxide stage 1, and chlorine dioxide stage 2. Each stage has specific functions, ranging from lignin separation, lignin removal, and whiteness improvement to final perfection [16].

Under the pressure of environmental protection, global changes have occurred in the bleaching process, including the emergence of natural color paper [17]. Eliminating the bleaching step can reduce or avoid chemical use, reduce costs, improve industry benefits, and eliminate dioxin generation (first-class carcinogens), rendering the paper healthier for the human body [18].

### 2.1. Raw Materials for Pulping

The composition of pulping raw materials is as follows: pulp is obtained from woody materials through physical, chemical, and biological pulping processes to extract fibers [19]. The pulping industry primarily utilizes fiber-rich woods as raw materials because their sufficient cellulose content meets the various needs of the industry [20].

Wood remains the primary raw material in the pulping industry [21]. However, several non-wood raw materials, such as hemp, wheat straw, cotton, bagasse, corn, and straw, can also serve as cellulose sources. The structural composition of wood and non-wood materials is considerably different due to factors such as their environment, age of growth, and geographical location (Table 1). Wood is categorized into hardwood and softwood. Among them, hardwood fibers are longer and the most suitable wood raw materials for pulping [22].

Cellulose, which is the most abundant polysaccharide substance present in plant cell walls, comprises two D-glucose molecules linked by β-1,4 bonds, and its average degree of polymerization can reach up to 10,000. Within cell wall microfibrils, cellulose is present in disordered (amorphous) and ordered (crystalline) structures [23]. Based on the cellulose chain arrangement and differing crystallinity of hydrogen bonds, cellulose is further divided into I, II, III, III II, IV I, and IV II [24]. Native cellulose exists as cellulose I with two crystal forms, Iα and Iβ, each with different hydrogen bonding patterns [25].

Hemicellulose, which is the second most abundant polysaccharide present in the plant cell wall, comprises two or more monosaccharides (such as arabinose, glucose, mannose, galactose, and xylose) that are linked together through covalent bonds, hydrogen bonds, and lipid bonds. Composed of links, the degree of aggregation is low, ranging from 70 to 200 [26]. The type of hemicellulose in a specific raw material depends on its fiber origin. Xylan with 1,4-β-D-xylopyranosyl units prevails in hardwood and agricultural biomass, constituting 20–37% of hardwood biomass [27]. Mannan or galactomannan is primarily present in coniferous wood raw materials, representing 16–27% of coniferous raw materials [28].

In addition, lignin, which is an aromatic, rigid, cross-branched, three-dimensional polyphenylene propane complex, is a vital cell component. It is heavier than cellulose and has a degree of polymerization between 50 and 500 [29]. In combination with hemicellulose, lignin forms a matrix that renders lignocellulose rigid and challenging to pulp [30]. It plays a crucial role in determining paper quality. Delignification from lignocellulosic biomass remains challenging, and the use of chemical methods to remove lignin affects the final pulp yield [31]. The pulping industry often alleviates costs by converting delignified waste into energy forms such as biogas, biofuel, or electric energy [32].

### 2.2. Other Ingredients

In addition to cellulose, hemicellulose, and lignin, plant tissue processing involves other elements, such as pectin and ash. Pectin, which is a type of polygalacturonic acid polysaccharide connected by α-1,4 bonds, is widely found in the primary and middle layers of plant cell walls. It is soluble in water but not readily soluble in organic solvents and exhibits good heat resistance. In pulping, pectinase pretreatment is commonly employed to remove pectin [33]. Ash, which is an inorganic substance that remains after physical and chemical transformations such as high temperature and combustion, affects the physical and mechanical properties of paper. Furthermore, the presence of SiO_2_ in the ash complicates alkali recovery.

#### 2.2.1. Wood Raw Material

**Figure 2:**
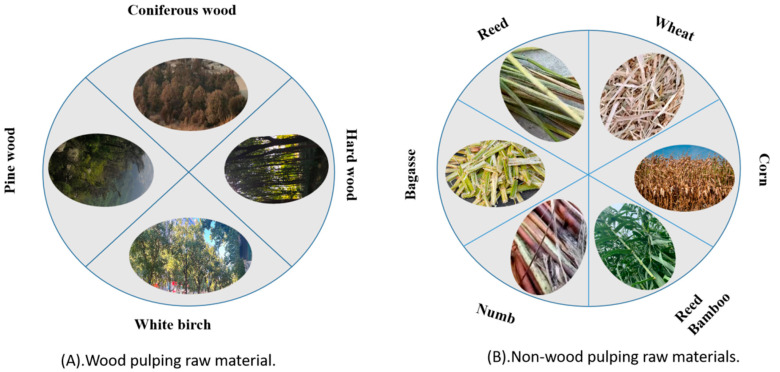
Different pulping raw materials.

Plant fiber is irreplaceable as the primary raw material for pulping and papermaking (Figure 2). Almost all plants can provide fiber, but not all are suitable for pulping and papermaking. Raw materials must be easily obtainable, abundant, inexpensive, and have suitable fiber lengths with minimal impurities. Wood species that are used for pulping include coniferous (pine, spruce, and fir) and hardwood species (such as eucalyptus, fir, poplar, and ash) [34].

Wood accounts for the largest proportion of papermaking material, offering a good fiber aspect ratio, minimal miscellaneous cell content, and higher pulping yield. Various types of wood are available throughout the year; however, not all wood types are suitable for pulping. Moreover, their suitability depends on their growth cycle, cultivation cost, processing complexity, and economic benefits. In general, the wood used is divided into the following two types: hardwood and coniferous wood. Hardwood pulp materials include eucalyptus, poplar, and birch. Tripathi et al. studied the influence of the presence of bark on the pulping effect of mixed hardwood pulp raw materials and found that an increase in bark ratio in raw wood chips led to more active alkali consumption, waste residue, and less pulp yield after kraft pulping [35]. Compared with the mixed hardwood without bark, paper pulp with bark exhibits lower tensile strength, burst resistance, and tear index, with decreases of over 58%. Compared to hardwood, softwood offers a longer fiber length, greater toughness, better stretchability, and high-quality paper production [36]. The most commonly used pine materials in the pulping industry are larch, *Pinus sylvestris* (*P. sylvestris*), Scotch pine, and Korean pine. Markus found that high-alkali low-temperature (HALT) polysulfide pulping can prevent the loss of the main polysaccharides while ensuring lignin removal. The wood yield of HALT pulp increased by 6.7 wt% compared to conventional pulp with a kappa number of 60 for comparable viscosity [37]. J. Petinarakis et al. explored the kraft pulping of wood samples from two cork pine provenances in northern Scotland with various concentrations of chemical reagents. Their study determined the relationship between different pulping conditions and the properties and quality of the pulp produced. The results indicated a cellulose loss of 17–28%, depending on the concentration of chemicals used in the cooking liquor. Using the kraft pulping process to explore the final pulping yield of *P. sylvestris* from inland to coastal areas revealed that the raw materials of *P. sylvestris* in different areas had different pulping effects [38]. When the same pulping raw materials are examined, the optimal pulping method and the concentration and volume of chemical and biological reagents must be determined through certain pre-experiments to obtain the most suitable pulping process. However, with increasing global demand from the pulping industry and issues surrounding environmental resources and the sustainable development of forests, increased restrictions have been implemented on the use of wood as a raw material. Non-wood raw materials, such as straw, and recycled fibers, and waste newspapers, are increasingly being developed for pulping [39].

#### 2.2.2. Non-Wood Fiber Raw Materials

Compared to wood fiber, non-wood fiber is predominantly used in countries with a limited wood supply. However, even in nonrestricted countries, the use of non-wood fiber materials for pulping is increasing [40]. Therefore, non-wood fibers have the potential for development as raw materials for pulping.

Non-wood raw materials for pulping primarily include waste crop straws and non-wood crops. Before being used as pulping raw material, a large amount of this waste straw is directly burned in the open land due to its low value and inconvenience in treatment, causing considerable smog production and substantial environmental damage. Around 3000 BC, the Egyptians were thought to have created the first writing material by gluing together thin fibrous stems for written records. However, this material is not considered a true form of paper because it lacks complete defibrillation (an essential process in papermaking) [41]. True papermaking was discovered in China in AD 105, where hemp rags and mulberry tree fibers were used to make paper. With ongoing advancements in pulping technology, pulping methods have evolved beyond purely chemical or mechanical methods. The emergence of biomechanical pulp has revitalized the use of waste crop straw as an essential pulping raw material. This prevents the pollution associated with annual incineration and provides a more suitable treatment plan for agricultural waste, thereby mitigating global environmental pollution and resource utilization issues [42].

Currently, the main waste crop straws being used as raw materials for pulping include corn stalks, wheat straw, bagasse, rice straw, coconut shell, bamboo, reeds, hemp, sorghum stalks, kenaf and other materials. This article discusses several of the most widespread and representative raw materials among non-wood raw materials, highlighting their advantages as raw materials and for the main pulping processes.

##### Corn Stalks

Corn is a primary food crop for humans; vast amounts of corn stalks are produced every year. Corn stalks were among the earliest non-wood raw materials used. Ferdous et al. examined the yield and papermaking effect of corn stalks in Kashi using different pulping methods. They found that the caustic soda–anthraquinone (AQ) method most easily removed lignin from the corn stover, resulting in the lowest pulp yield and kappa number. These pulps exhibit a good tensile index (75–85 Nm/g) and a moderate tear index (4.5–6 mN/m^2^/g) after being formed into paper [43]. Most non-wood materials contain considerable amounts of SiO_2_, which can foul the digester and complicate lime mud filtration and subsequent recycling [44]. Research has revealed that organic acid pulping is favorable for the delignification process of crop residues, leaving SiO_2_ on pulp fibers [45]. Furthermore, organic acid pulps can be bleached without the use of chlorine [45].

Mishra et al. investigated the composition, pulping, bleaching performance, and physical strength of corn stalks. They revealed that the alkali solubility of corn stalks is 41.8%, with a composition of acetone extracts at 3.5%, cellulose at 53.6%, hemicellulose at 26.4%, and Klassen lignin at 20.0% [46]. To produce pulp with a kappa number of 16.1, 14.5% alkali (NaOH) is required, resulting in a pulp yield of 53.7% [46]. A comparison between the paper strength of bleached and unbleached pulp revealed that the physical strength (particularly the tensile index) of bleached pulp surpasses that of unbleached pulp. This discrepancy may arise from the interference of residual lignin in the fibrillation process, thus reducing paper strength. The steam explosion process performed by Xia et al. was adopted, and the optimal process conditions were determined as follows: pretreatment with 2% sulfuric acid, an immersion time of 30 min, a steam explosion pressure of 1.6 MPa, and an explosion time of 5 min. Under this process, a hand sheet with a weight of 80 gm^−2^ was produced through sandblasting, with a burst index of 0.99 kPam^2^g^−1^, a tensile index of 24 nmg^−1^, and a ring pressure index of 0.58 nMG^−1^. These values are close to the strength index of corrugated paper [47]. Using this method to bio-refine corn stalks and produce pulp is an industrially promising solution, facilitating the high-value recycling of waste straws.

##### Wheat Straw

Wheat straw, which is more widely grown than corn stover, was earlier among the first non-wood fibers used in the pulp and paper industry. Decades ago, wheat was extensively utilized as a pulping raw material in major factories. It was later abandoned, mainly because pure chemical pulp was less profitable than wood, and papermaking with black liquor was more challenging to process and remove. The presence of silicon presented even greater interference during alkali recovery [48]. However, with the development of biopulping, wheat straw has regained value within the pulping industry. Biopulping, incorporating microorganisms and enzymes, offers a cleaner and greener method for pulping.

M. S. Jahan primarily researched the separation of SiO_2_ and pulp from rice straw by pulping black liquor using the potassium hydroxide (KOH) method [49]. They revealed that the optimal conditions for rice straw pulping include an alkali dosage of 12%, a cooking temperature of 150 °C, a cooking time of 2 h, and a solid-to-liquid ratio of 1:6, resulting in a pulp yield of 42.4% and a kappa value of 10.3. The KOH pulp was bleached to 85% brightness using the D0Epd1 bleaching program, consuming 25 kg of ClO_2_ per t of pulp.

Varghese et al. investigated the feasibility of using xylose–pectin hydrolase in wheat straw pulping. They reported the optimal dosage of composite enzymes to be 40,000 xylanase and 12,000 IU/kg of pectinase at a material-to-liquid ratio of 1:10 (g/mL), a temperature of 55 °C, and an enzyme soaking time of 3 h in 0.75% Tween 80 (pH 8.5). Enzyme pretreatment achieves a considerably higher pulp performance than chemical pulping, with a higher pulp yield, lower kappa number (15.67%), and lower residual rate (59.65%) [50]. Compared to pure chemical pulping, pretreatment with biological enzymes effectively improves pulp properties in straw pulping. Biological pulping reduces chemical components by 12%, increases residual alkali, and attains optical and chemical properties similar to purely chemically treated pulp. In addition, the paper strength of the pulp after enzyme treatment was improved, increasing the burst index, tear index, and breaking length. Pulp, when treated with enzyme and chemical reagents, demonstrated increased brightness and decreased yellowness at all bleaching stages [51]. These findings indicate that enzyme pretreatment enhances the efficiency of the caustic soda–AQ pulping of wheat straw reduces lye consumption, produces less harmful wastewater, and improves paper quality. Therefore, this method presents a dual-benefit solution that can be adopted by the pulping process and the industry.

##### Bagasse

Bagasse, which is a byproduct of sugar cane obtained after it is squeezed, is a widely used pulp raw material. Sugar cane, which is the main raw material crop for sucrose production, is extensively grown in various countries, including Brazil, China, and India, with exports from more than 100 countries worldwide. The bagasse paper industry has emerged to utilize this significant byproduct.

Yue et al. investigated the low-temperature oxygen–alkali pulping of bagasse, and the optimal process conditions were determined as follows: pulping at 100 °C, an active alkali dosage of 23%, a highest cooking temperature of 100 °C, a holding time of 180 min, and an initial oxygen pressure of 0.6 MPa. In addition, they used a magnesium sulfate dosage of 0.5% and a bagasse concentration of 12%. The yield of the obtained pulp was 60.9%, the kappa number was 14, the paper viscosity was 766 dm^3^/kg, and the whiteness was 63.7% [52]. This process had the lowest cooking temperature reported thus far and is a more environmentally friendly and energy-saving pulping process.

##### Rice Straw

The process of straw pulping is similar to that of wheat straw pulping. Rice straw is also one of the world’s largest sources of renewable agricultural residual waste. Approximately 800 million t of rice straw are produced globally each year. Rice straw is not only rich in cellulose and hemicellulose but also contains ~10% ash, of which ~75% is silica. The fiber length of rice straw is comparable to that of hardwood raw materials, and the hemicellulose content is also similar [53]. Zhou et al. investigated the influence of aerobic fermentation pretreatment using complex bacterial agents on rice straw biomechanical pulping. During aerobic fermentation, they detected parameters such as microbial community, chemical composition, and surface morphology in different fermentation cycles. By optimizing biomechanical pulping based on the properties of the pulp beating degree, energy consumption, water retention, and drainage value, they found that under optimal conditions, pre-fermentation treatment can reduce energy consumption by ~54%, increase WRV by 13%, and enhance tensile strength by 81% at a given pulp freeness [54].

##### Coconut Shell

Coconut shells possess a high cellulose content (up to 50%) and an extremely low ash content (~0.7%), rendering it a suitable raw material for pulping [55]. Faisal RM Afrifah explored using coconut leaves to produce art paper. The investigations revealed that coconut leaves contain a considerable amount of fiber and cellulose. To avoid waste and protect the forest environment, chemical pulping methods were utilized, and then the tensile strength of the prepared art paper was tested. The created art paper samples achieved 100 g/m^2^. For the 30/75 sample, the optimal draw resistance was ~19,800, whereas for the 60/75 sample, the best tensile resistance test result was 0.955 [56].

##### Bamboo

Bamboo belongs to the tall tree-like grass plant family, with fast growth, relatively hard stems, and a woody hollow structure. The cellulose content in bamboo is 40–50%, which is situated between softwood and hardwood. Cellulose is an excellent raw material for pulping [57]. Liu et al. utilized cocultured microorganisms comprising environmental microorganisms and Bacillus and found that bamboo pulp to pretreat bamboo enhanced the pulping performance and reduced consumption; the removal rates were 21.96% and 26.21%, respectively, and the yield of bamboo pulp increased [58]. This method aligns more closely with the current environmental protection requirements and achieves the goal of biorefining.

Gao et al. used parenchyma cellulose from the large-scale clustered bamboo unique to Yunnan, China, to prepare regenerated cellulose films. Bamboo parenchyma cells were isolated from low-temperature (maximum temperature: 140 °C) kraft slurry through multiple screenings. Cellulose was dissolved in a N,N-dimethylacetamide (DMAc) solvent with a LiCl content of 6–10% and then regenerated in a glycerol–water solution. X-ray diffraction analysis revealed that the crystalline form of the RC film transitioned from cellulose I to cellulose II [59]. A lower amount of LiCl is beneficial for enhancing the thermal stability of the RC film, and it exhibits a strong cellulose-dissolving ability, demonstrating its significant industrial application prospects, which are conducive to the high value-added utilization of waste biomass resources.

##### Reeds

Phragmites reed is an aquatic, wet, and tall herbaceous plant of the grass family, which is often found along rivers worldwide. Raimo et al. conducted orthogonal experiments in caustic soda–AQ pulping, considering four factors and three levels. They explored the effects that the number of chemical reagents used in reed raw materials had with a maximum temperature (145 °C, 155 °C, and 165 °C), time to reach the maximum temperature (70, 90, and 110 min), and maximum temperature holding time (0, 15, and 30 min) on the pulp yield, calorific value, primary value, and viscosity. The results indicate that all four factors considerably affect pulp viscosity, except for the time to reach the highest temperature. For lignification removal, alkali concentration emerged as the most critical parameter, whereas other parameters exerted little effect on delignification [60].

##### Hemp

Hemp, which is one of the most easily available long and soft natural fibers, can be woven into high-strength coarse filaments and utilized as raw materials in the textile and papermaking industries. Jahan et al. [61] studied the effect of band degumming on kraft pulping. The results revealed higher pulp yields for conventional jute fibers and jute sticks at any kappa number. The paper properties of the prepared pulp were not considerably affected during the degumming process. However, the bleaching performance of jute fiber pulp was better than that of jute stick pulp. Compared with other non-wood raw materials, hemp is predominantly used as a textile material.

##### Sorghum Stalks

Sorghum, which is an annual herbaceous plant in the Poaceae family, has strong stalks and a good growth cycle, rendering it a viable source of pulping raw materials. Alves et al. optimized sorghum straw pulping and bleaching processes using centralized composite experiments and investigated the following four factors: cooking time, dilute alkali concentration, bleaching time, and bleaching volume. The study found that the optimal processing conditions were 2.5 h of cooking time, 1.25% of a dilute alkali concentration at 90 °C, 35 min of bleaching time, and 25 mL of the bleaching volume at 80 °C. Under these conditions, a pulp with a kappa value of less than three and low lignin content, as well as a fiber with a high degree of crystallinity, could be obtained [62].

##### Kenaf

Kenaf is also known as Hibiscus cannabinus. It is an important fiber resource for the production of sacks, carpets, and twine. It is easy to plant and grow, has a high yield, has a wide range of sources, is rich in resources, has excellent moisture absorption and air permeability, and also has good mechanical properties. As a non-wood raw material, the fiber content of kenaf is similar to that of wood with less lignin. As a papermaking raw material, the pulping yield is about 50%. It can be used as an excellent source of pulp. The pulping properties of kenaf at different growth stages were studied by Saffet Karakus et al. The results show that, when harvested at about 150 days of growth, the yield is the highest, and at the same time, the lignin content of the raw material is the lowest [63]. Alireza Ashori and others used different polymers as sizing agents on the basis of kenaf pulping to improve the surface morphology of the paper. The results show that the paper properties were improved, and the surface was smoother under the treatment of three polymers, among which chitosan had the most obvious improvement effect [64]. The physical properties of kenaf paper are improved through additive treatment, providing new solutions for improving the performance of kenaf and other non-papermaking raw materials.

##### Other Materials

In addition to natural plant materials, wastepaper, and newspapers are valuable raw materials for pulping. Refinement after de-inking can rejuvenate wastepaper, giving it a new appearance and life [65]. Bamboo reed, which can be extensively planted in saline–alkali land, offers further potential. It not only aids in controlling saline–alkali terrain but also grows quickly. After reaching maturity, the stems that survive the winter possess fibers with a good aspect ratio, rendering them suitable as raw materials for pulp and paper. In addition, non-plant fibers have been explored as raw materials for pulping. To alleviate the increasing global environmental problems and the growing shortage of wood resources in the pulping industry, researchers have developed new materials for pulping. Cellulose synthesized by microorganisms has emerged as a promising new material [66]. Research in this area mainly focuses on innovative materials such as nanocellulose and composites and has expanded in recent years.

## 3. Pulping Process

### 3.1. Traditional Pulping Process

Traditional pulping processes are mainly mechanical and chemical [67]. Mechanical pulping has been used since the beginning of the earliest papermaking technology. The pulping of raw materials was beaten and crushed to obtain the internal fibers and then used for papermaking. This method has been developed to the present time, and the equipment has been continuously upgraded. Disk refiners and refiners are used, increasing the energy consumption of the pulping process. However, the performance of the pulp has been considerably improved, and the yield of this pulp is high [68].

Compared with mechanical pulping, the chemical pulping process uses chemicals to destroy the surface of pulping raw materials at a certain temperature and pressure, penetrating the interior and destroying lignin and some nonfibrous carbohydrates as well as pectin substances [69]. The most commonly used chemical methods are the sulfate and sulfite methods. Gulsoy studied the pulping process by combining biological enzyme preparation and the kraft method. The results revealed that this material, after biological enzyme pretreatment, changed with time, and paper strength exhibited an irregular downward trend [70]. Among them, the sulfate method is more commonly used than the sulfite method because of its most perfected alkali recovery process and the effective treatment of pulping pollutants. In addition, there are more environmentally friendly and energy-saving pulping processes, such as oxygen–alkali pulping and alkaline H_2_O_2_ pulping [71].

### 3.2. Biopulping

Biopulping uses biological enzyme preparations, such as white rot fungal agents, that degrade lignin, cellulase, and pectinase. The biological agent is treated for a certain period before pulping to reduce energy consumption and the use of chemical reagents, which is more in line with the requirements of environmental protection [72]. Nagpal et al. used pectinase and xylanase as the research objects to explore biological pulping with rice straw as the raw material. They found that, compared with chemical pulping, the yield and whiteness of the obtained pulp were improved. In addition, they replaced 10% of the alkali dosage [73]. Therefore, with the development of society, people’s requirements for environmental protection are increasing, and the biopulping process receives increasing attention in the pulping industry [74].

### 3.3. Other Pulping Methods

In addition to the commonly used mechanical, chemical, and biological pulping processes, new pulping processes are being constantly discovered [75]. In supercritical ammonia pulping, supercritical ammonia is used as a fluid to extract lignin from pulping raw materials. Ammonium sulfite pulping uses ammonium sulfite as a lignin removal agent. These innovative methods promote the pulping industry to become more energy-efficient and environmentally friendly [76].

The analyses of the pulping process of the abovementioned raw materials, the effect of pulping, and physical and chemical indicators reveal that under the current pressure of resources and environmental protection, comparing woody raw materials with high resource and energy consumption, non-woody raw materials are increasingly valued by the pulp and paper industry. Thus, non-wood and recycled fibers have considerable potential for replacing wood fibers [77]. Currently, the pulping process that is most suitable for the factory is being explored through various research experiments. Compared with wood raw materials, using non-wood materials for pulping has more advantages, such as the easy availability of raw materials, a higher quality of pulp after bleaching, the multiple sources of raw materials, and growth cycle segments, which can considerably reduce resources and energy consumption wastage, protect the environment and better promote its green and healthy development [78].

Combined with the problems faced by the current pulping industry, it is necessary to continuously develop non-wood as raw materials for pulp and paper [79]. Using these non-wood materials can address the issue of an insufficient supply of wood due to environmental protection requirements, increase the utilization of agricultural wastes, industrial raw materials, and waste paper, increase production, and better protect the environment.

## 4. Summary and Outlook

With the development of society, the products of the pulping industry, especially paper products, have entered into all aspects of people’s lives and played an increasingly important role in people’s lives. At the same time, people’s demand for high-quality paper products has also greatly promoted the development of the industry. As the main raw material for pulping, wood has a slow growth cycle and logging regulatory restrictions. The gradually shrinking supply cannot meet the rising demand. Herbs, which also contain a large amount of fiber, could become an important choice for pulping raw materials in the future due to their fast growth, large reserves, and easy availability. In the context of today’s rapid development of biomodification technology, it is increasingly convenient to develop herbal plants that are suitable for the pulping industry. It is expected that more plant raw materials with a good fiber length, high yield, and suitable for paper making will be used in the future. At the same time, man-made fiber technology is developing rapidly, and man-made fibers represented by viscose fiber, acetate fiber, and cupro fiber are also expected to become an important source of raw materials for pulping in the future.

Not only is wood subject to many limitations as a raw material for pulping, but traditional pulping methods that are extensive, high in alkali, and have a high dosage of chemicals are also being gradually phased out due to shortcomings such as high pollution, high energy consumption, and high costs. As early as more than 2000 years ago, people created the earliest paper by using natural bacteria to decay raw materials without using chemicals. With the vigorous development of biotechnology, microorganisms have been modified and used in various industries. In the pulping industry, biological agents such as bacterial strains and enzymes that are efficient and suitable for the pulping environment are constantly being discovered and utilized. Due to their advantages, such as mild use conditions, high efficiency, and specific recognition sites, biological agents can reduce energy consumption in the pulping production process, reduce the emission of pollutants later, and save pulp production costs.

In the future, the pulping industry will not only need to develop known non-wood raw materials to improve the physical properties of its pulp but also continue to look for paper-making raw materials that are comparable in strength to wood raw materials and are easier to obtain. At the same time, with the support of biotechnology, biological agents that are suitable for various environments can be transformed. We believe that in the future, with the application of new technologies and new processes, chemical pulping technology and biological pulping can be perfectly combined, making the pulping and papermaking production process more environmentally friendly and efficient so that the pulping industry can develop more vigorously.

## Figures and Tables

**Table 1:** Main chemical composition of pulping raw materials (adapted from Drake Mboowa [1]).

Raw Materials	Cellulose (%)	Hemicellulose (%)	Lignin (%)
Hardwood	43–47	25–35	16–24
Coniferous Wood	42–50	24–34	15–22
Bagasse	40	30	30
Corn stalk	37	25	35
Wheat	43.7	23	22.3
Rice	43	25	16
Cizhu	46	32	12
Reed Bamboo	48	26	18
